# Diagnosis of Lung Cancer by FTIR Spectroscopy Combined With Raman Spectroscopy Based on Data Fusion and Wavelet Transform

**DOI:** 10.3389/fchem.2022.810837

**Published:** 2022-01-26

**Authors:** Xien Yang, Zhongyu Wu, Quanhong Ou, Kai Qian, Liqin Jiang, Weiye Yang, Youming Shi, Gang Liu

**Affiliations:** ^1^ Yunnan Key Laboratory of Opto-electronic Information Technology, School of Physics and Electronic Information, Yunnan Normal University, Kunming, China; ^2^ Department of Thoracic Surgery, The First People’s Hospital of Yunnan Province, Kunming, China; ^3^ School of Preclinical Medicine, Zunyi Medical University, Zunyi, China; ^4^ School of Physics and Electronic Engineering, Qujing Normal University, Qujing, China

**Keywords:** lung cancer, FTIR spectroscopy, Raman spectroscopy, data fusion, wavelet transform

## Abstract

Lung cancer is a fatal tumor threatening human health. It is of great significance to explore a diagnostic method with wide application range, high specificity, and high sensitivity for the detection of lung cancer. In this study, data fusion and wavelet transform were used in combination with Fourier transform infrared (FTIR) spectroscopy and Raman spectroscopy to study the serum samples of patients with lung cancer and healthy people. The Raman spectra of serum samples can provide more biological information than the FTIR spectra of serum samples. After selecting the optimal wavelet parameters for wavelet threshold denoising (WTD) of spectral data, the partial least squares–discriminant analysis (PLS-DA) model showed 93.41% accuracy, 96.08% specificity, and 90% sensitivity for the fusion data processed by WTD in the prediction set. The results showed that the combination of FTIR spectroscopy and Raman spectroscopy based on data fusion and wavelet transform can effectively diagnose patients with lung cancer, and it is expected to be applied to clinical screening and diagnosis in the future.

## Introduction

Lung cancer is a malignant tumor with a high incidence rate and a high mortality rate threatening human health (Sung et al., 2021). Due to the lack of biomarkers in lung cancer, most patients are in the middle and advanced stage at the time of treatment (Stapelfeld et al., 2020). At present, the screening methods of lung cancer mainly include X-ray examination, low-dose computed tomography, and magnetic resonance imaging (MRI), but these technologies have some disadvantages, such as unable to apply to specific populations, high false positive rate, and low sensitivity (Thakur et al., 2020; Xu et al., 2021). Therefore, there is a need to find an early diagnostic method with wide application range, high specificity, and high sensitivity.

Vibrational spectroscopy is an important tool in the field of analytical chemistry and bioanalysis, of which Fourier transform infrared (FTIR) spectroscopy and Raman spectroscopy have been widely used in cancer diagnosis in recent years (Auner et al., 2018; Christensen et al., 2019; Baiz et al., 2020). In our previous work, we studied the serum samples of patients with lung cancer and healthy people using FTIR spectroscopy and found that the concentrations of protein, lipid, and nucleic acid molecules in the serum of patients with lung cancer were higher than those of healthy people (Yang et al., 2021a). Song et al. classified the tissues of healthy people and patients with lung squamous cell carcinoma using Raman spectroscopy combined with principal component analysis–linear discriminant analysis (PCA-LDA) (Song et al., 2020). These reports demonstrate the potential of FTIR spectroscopy and Raman spectroscopy in the diagnosis of lung cancer.

Data fusion has been widely used in the analysis and determination of biological and pharmaceutical components in recent years because of its integration of multiple methods to obtain more effective and comprehensive data (Haware et al., 2011; Comino et al., 2018; Feng et al., 2020; Zhang et al., 2020; Zhao et al., 2020; Azcarate et al., 2021). There are reports that data fusion was used in FTIR spectroscopy and Raman spectroscopy for the diagnosis of thyroid dysfunction and cervical cancer. Chen et al. studied the blood of patients with thyroid dysfunction and healthy people using FTIR spectroscopy and Raman spectroscopy combined with data fusion and achieved an accuracy of 83.48% (Chen et al., 2020). Zhang et al. studied the tissue samples from patients with cervical cancer using Raman spectroscopy and obtained an accuracy of 93.51% using characteristic data after fusion of first and second derivatives (Zhang et al., 2021). Therefore, data fusion combined with FTIR spectroscopy and Raman spectroscopy has the potential to diagnose various diseases, so it is expected to be applied in the diagnosis of lung cancer.

As a powerful signal processing technology, wavelet transform has been widely used in imaging, chromatography, vibration spectroscopy, and so on (Sudarshan et al., 2016; Jiang and Ma, 2020; Wahab and O’Haver, 2020; Sun et al., 2017; Godinho et al., 2014; Martyna et al., 2015; Dinç and Yazan, 2018). There are reports that wavelet transform and data fusion were used in combination with some other techniques to detect prostate cancer and neurocysticercosis. Tiwari et al. fused the data of magnetic resonance (MR), imaging (MRI), and spectroscopy (MRS) using multimodal wavelets (MaWERiC) and found that the MaWERiC had better detection results for prostate cancer than any single data (Tiwari et al., 2012). Chavan et al. proposed a non-subsampled rotated complex wavelet transform (NSRCxWT) extraction image fusion algorithm based on computed tomography (CT) and MRI features and found that the image quality processed under this algorithm was much better than the original image quality, which was more conducive to the diagnosis of neurocysticercosis (Chavan et al., 2017). However, the method of combining wavelet transform and data fusion using FTIR spectroscopy and Raman spectroscopy has not been studied in the diagnosis of lung cancer.

In this study, data fusion and wavelet transform were used in combination with FTIR spectroscopy and Raman spectroscopy to make full use of the FTIR and Raman spectral information of serum samples, and then distinguish the serum of patients with lung cancer from that of healthy people. The purpose is to explore a wide applicable and high-accuracy diagnostic method for lung cancer and to lay the foundation for the clinical application of FTIR spectroscopy and Raman spectroscopy in the diagnosis of lung cancer in the future.

## Materials and Methods

### Serum Samples

Serum samples from 92 patients with lung cancer and 155 samples from healthy people were obtained from The First People’s Hospital of Yunnan Province, and all research content was conducted according to the Declaration of Helsinki.

### Spectra Acquisition

FTIR spectra of serum were measured in the range of 4000–600 cm^−1^ by a Frontier spectrometer (Perkin Elmer) with the same test method as in our previous work (Yang et al., 2021a). Each IR spectrum was an accumulation of 32 scans at a resolution of 4 cm^−1^. Serum samples (30 μl) were dropped on clean glass slides to measure Raman spectra using a confocal micro Raman spectrometer (ANDOR SR-500-type) in the range of 800–1800 cm^−1^ through a ×50 objective lens with an excitation wavelength of 532 nm for the laser. The laser power at the serum sample was 12 mW. Each Raman spectrum was scanned for 10 s and accumulated three times.

### Wavelet Threshold Denoising

Wavelet transform is the projection of signal on a wavelet base (Rameshnath and Bora, 2019). It gradually refines the signal in multiple-scale through expansion and translation operations and, finally, realizes the time subdivision of high frequency and the frequency subdivision of low frequency so as to focus any details of the signal. In the wavelet domain, the wavelet coefficient of the signal is larger than that of the noise. The basic principle of wavelet threshold denoising is to set an appropriate threshold. The wavelet coefficients larger than the threshold are considered to be generated by the signal and should be preserved. Those smaller than the threshold are considered to be generated by noise and set to zero, thus achieving the purpose of denoising (Donoho and Johnstone, 1995). Wavelet denoising removes noise and maintains the details of the signal using multi-scale and multi-resolution characteristics of wavelet transform. Compared with the low-pass filter based on Fourier transform, wavelet denoising has a better effect (Peng et al., 2021).

The effect of the WTD algorithm on FTIR and Raman spectral data mainly depends on the optimal wavelet function, wavelet decomposition level (DL), and wavelet threshold. Choosing an appropriate wavelet function is helpful to maximize the coefficient value in the wavelet domain. Generally, the appropriate wavelet function is determined by the specific practical requirements (Liu et al., 2021). In wavelet decomposition, the choice of DL is also a very important step. The larger the DL is, the more obvious the characteristics of noise and signal are, which is more conducive to the separation of them. Unfortunately, the larger the number of DL is, the greater the distortion of the reconstructed signal is. The selection of threshold is divided into two parts: the selection of threshold function and the selection of threshold. The commonly used threshold functions are mainly soft threshold function and hardness threshold function. The result of the soft threshold function is smoother than the hard threshold, so the soft threshold function was selected (Sanam and Shahnaz, 2013).

### Spectral Data Preprocessing

In the process of spectral measurement, there are some inevitable interference factors, such as background disturbance, light scattering, and particle size, which influence the quality of raw spectra and decrease the accuracy of classification models (Chen et al., 2004). Therefore, several different preprocessing methods were used in order to reduce the unnecessary signal variations, such as normalization, Savitzky–Golay (SG) filter, first derivative (FD), second derivative (SD), and standard normal variate (SNV) (Roy, 2015; Everard et al., 2016).

### Data Fusion

Date fusion is the process of integrating data from different sources. The main purpose of data fusion is to find more valuable data set, which might improve the accuracy of prediction and present a better interpretation of the results (Li et al., 2021). In this study, matrices of FTIR and Raman spectral data were integrated into a single matrix. The FTIR matrix and Raman matrix were concatenated on the column forming a two-dimensional merged data matrix that has the same rows with the analyzed samples.

### Partial Least Squares–Discriminant Analysis

PLS-DA is a linear pattern classification method, which is widely used to deal with complicated data by reducing dimension. In this study, all samples were divided into a calibration set (60%) and a prediction set (40%) by the Kennard–Stone algorithm. Samples of patients with lung cancer were coded 1, while those of healthy people were coded 2, and the discriminant threshold of the model was set to 1.5. The performance of the PLS-DA model was evaluated in terms of accuracy, specificity, and sensitivity of calibration (Acc_cv_, Spe_cv_, and Sen_cv_) and prediction (Acc_p_, Spe_p_, and Sen_p_) (Yang et al., 2021b). The PLS-DA model, spectral data preprocessing, and WTD algorithm were performed using the MATLAB software (version R2019a, MathWorks).

## Results and Discussion

### Fourier Transform Infrared Spectra and Raman Spectra of Serum

**FIGURE 1:**
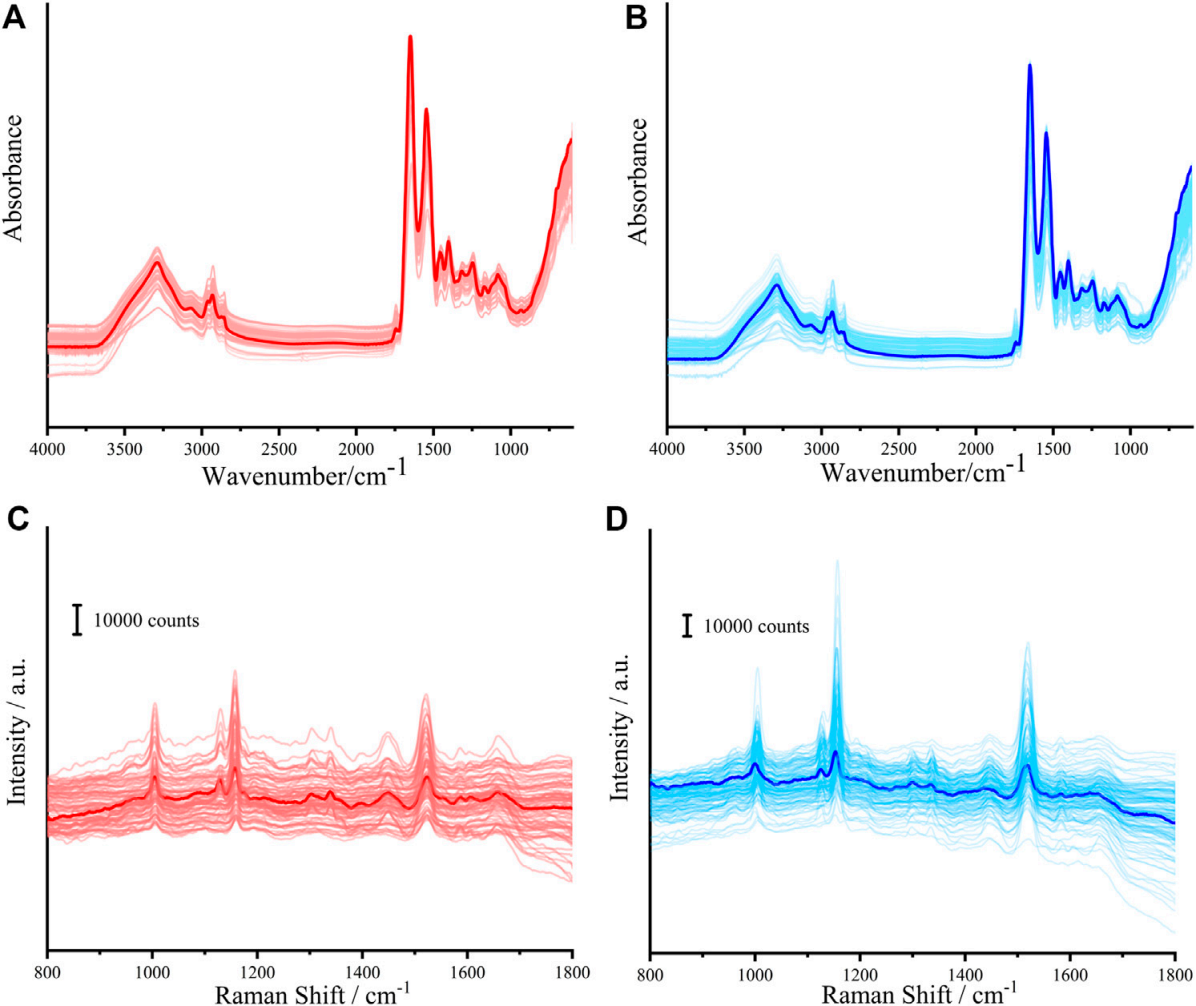
FTIR spectra of serum from patients with lung cancer **(A)** and healthy people **(B)**. Raman spectra of serum from patients with lung cancer **(C)** and healthy people **(D)**. (The corresponding average spectra are shown in bold).

Figure 1 shows the FTIR spectra and Raman spectra of serum samples. The main peaks and their assignments are listed in Table 1. It can be found from Table 1 that the Raman spectra of serum samples can provide more biological information than the FTIR spectra of serum samples, such as porphyrin, phospholipids, and glucose. It can be seen from Figure 1A and Figure 1B that the IR spectra of serum from patients with lung cancer are extremely similar to that of healthy people. Figure 1C and Figure 1D show the Raman spectra of serum from patients with lung cancer and that of healthy people, respectively. Although some differences between the patients with lung cancer and healthy people can be seen from the original Raman spectra of serum samples, it is also necessary to optimize and process the original FTIR and Raman spectral data to distinguish them.

**TABLE 1:** Peaks of FTIR and Raman spectra and their assignments.

Wavenumber/Raman shift	Peak assignments	References
Wavenumber (cm^−1^)	—	—
2,959	C-H asymmetric stretching vibration of CH_3_ in lipid	—
2,930	C-H asymmetric stretching vibration of CH_2_ in lipid	—
1740	C=O stretching vibration from ester carbonyl in triglycerides	—
1,646	α-helix structure in amide I protein	—
1,542	N-H functional group in amide II protein	—
1,243	P=O asymmetric stretching vibration of PO_2_^−^ in nucleic acids	—
1,079	P=O symmetric stretching vibration of PO_2_^−^ in nucleic acids	Yang et al. (2021a)
Raman shift (cm^−1^)	—	—
1,005	Symmetric ring breathing mode in phenylalanine, CHO, and protein	Bahreini et al. (2019)
1,129	C-N stretching in protein	Lakshmi et al. (2002); Chan et al. (2006)
1,155	C-C stretching in glucose, CHO, and protein	Bahreini et al. (2019)
1,302	C-H vibration in triglycerides	Silveira et al. (2002)
1,448	CH_3_-CH_2_ bending of phospholipids and the protein side chains	Yan et al. (2020)
1,520	C=C stretching in porphyrin	Movasaghi et al. (2007)
1,656	C=C stretching in lipid and amide I protein	Cheng et al. (2020)

### Model Performances for Spectral Data Processed by Wavelet Threshold Denoising

In order to optimize the FTIR and Raman spectral data to improve the classification effect of the PLS-DA model, ten commonly used wavelet functions, bior2.2, coif1, coif3, db02, db08, fk4, fk8, haar, sym5, and sym8, were tested. Each wavelet function was performed under 1–8 wavelet DLs to study the effect of DL on the denoising effect (Liu et al., 2021). At the same time, four threshold acquisition methods, heursure, minimaxi, rigrsure, and sqtwolog, were performed to further improve the denoising performance. The optimal values of wavelet function, DL, and wavelet threshold were determined by calculating the accuracy (Acc_cv_), specificity (Spe_cv_), and sensitivity (Sen_cv_) at 7-fold cross-validation by the PLS-DA model.

#### Model Performances for Fourier Transform Infrared Spectral Data Processed by Wavelet Threshold Denoising

**FIGURE 2:**
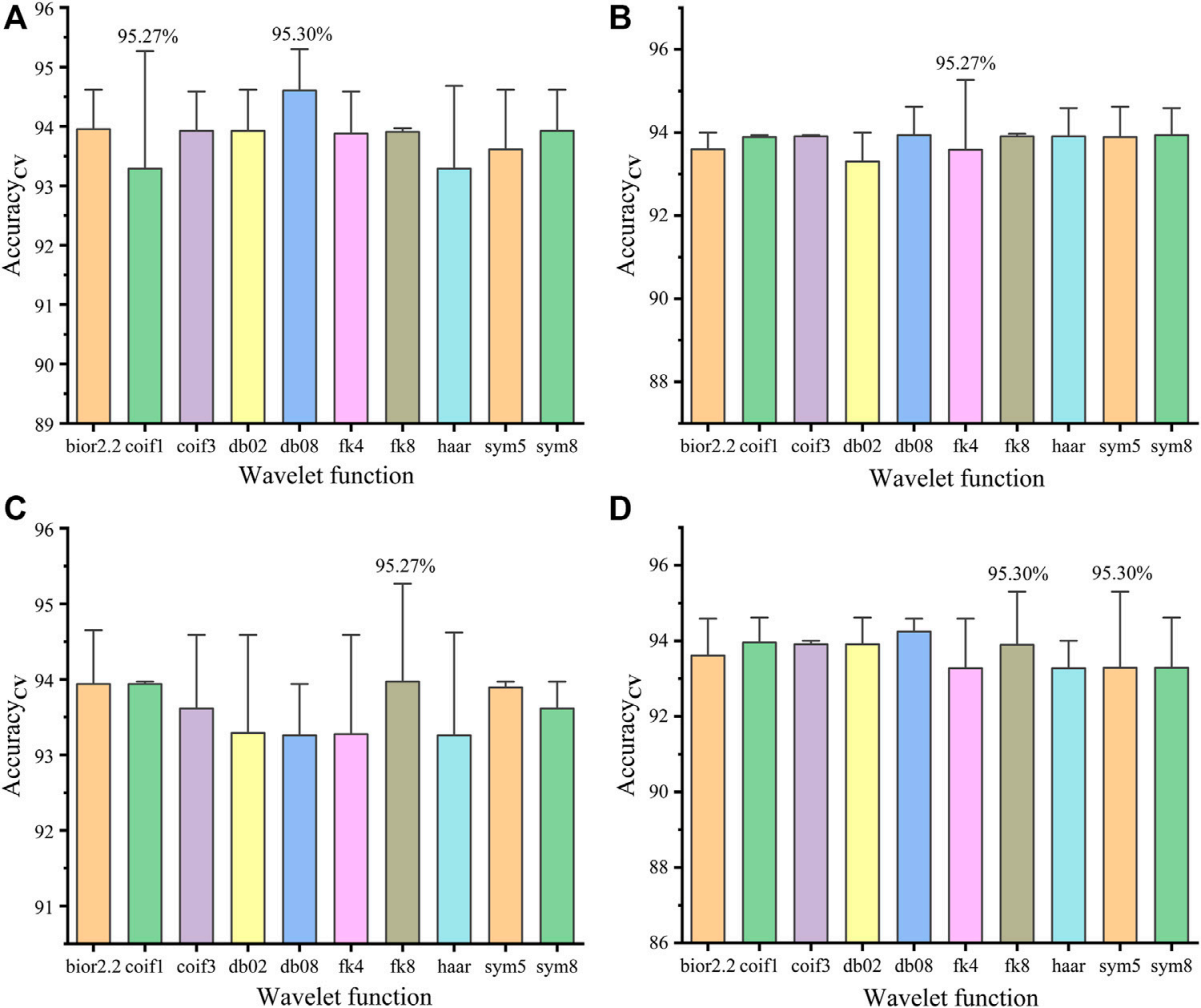
Acc_cv_ of the PLS-DA model using FTIR spectral data processed by WTD in four thresholds: heursure **(A)**, minimaxi **(B)**, rigrsure **(C)**, and sqtwolog **(D)**.

Figure 2 shows the Acc_cv_ (mean value + error bar) of the PLS-DA model using FTIR spectral data processed by WTD in four thresholds. It is shown that the combination of different thresholds and wavelet functions has different effects when processing the same FTIR spectral data. Where the combination of heursure and db08 (heursure-db08) has the same and highest Acc_cv_ as the combination of sqtwolog and fk8 (sqtwolog-fk8), but heursure-db08 has higher Sen_cv_ than sqtwolog-fk8 (Table 2). Figure 4 shows the choice of the best DL, where DL = 6 has the best performance for WTD (heursure-db08) of FITR spectral data. Therefore, heursure-db08 and DL = 6 were selected as the optimal wavelet parameters for WTD of FITR spectral data.

**TABLE 2:** Calibration results for FTIR spectral data processed by different WTD algorithms. (The best threshold, wave function, and DL for the PLS-DA model are presented in bold).

Threshold	Wave function	DL	Calibration		
			Acc_cv_ (%)	Spe_cv_ (%)	Sen_cv_ (%)
**heursure**	**db08**	**6**	**95.30**	**96.76**	**92.36**
minimaxi	fk4	7	95.27	99.05	88.75
rigrsure	fk8	8	95.27	98.81	85.12
sqtwolog	fk8	5	95.30	98.21	90.79

The meaning of the bold values is the best processing method for PLS-DA model.

#### Model Performances for Raman Spectral Data Processed by Wavelet Threshold Denoising

**FIGURE 3:**
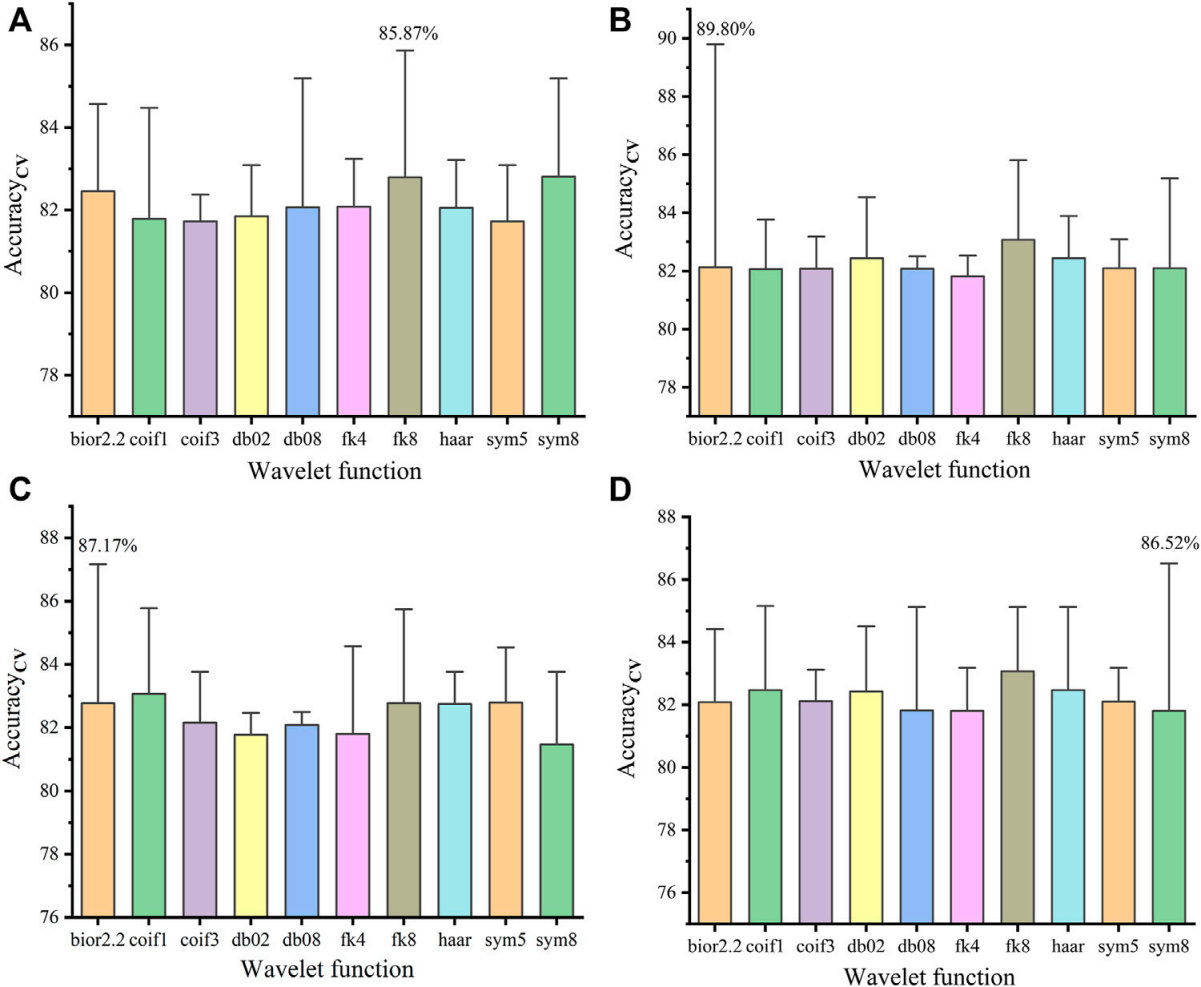
Acc_cv_ of the PLS-DA model using Raman spectral data processed by WTD in four thresholds: heursure **(A)**, minimaxi **(B)**, rigrsure **(C)**, and sqtwolog **(D)**.

**FIGURE 4:**
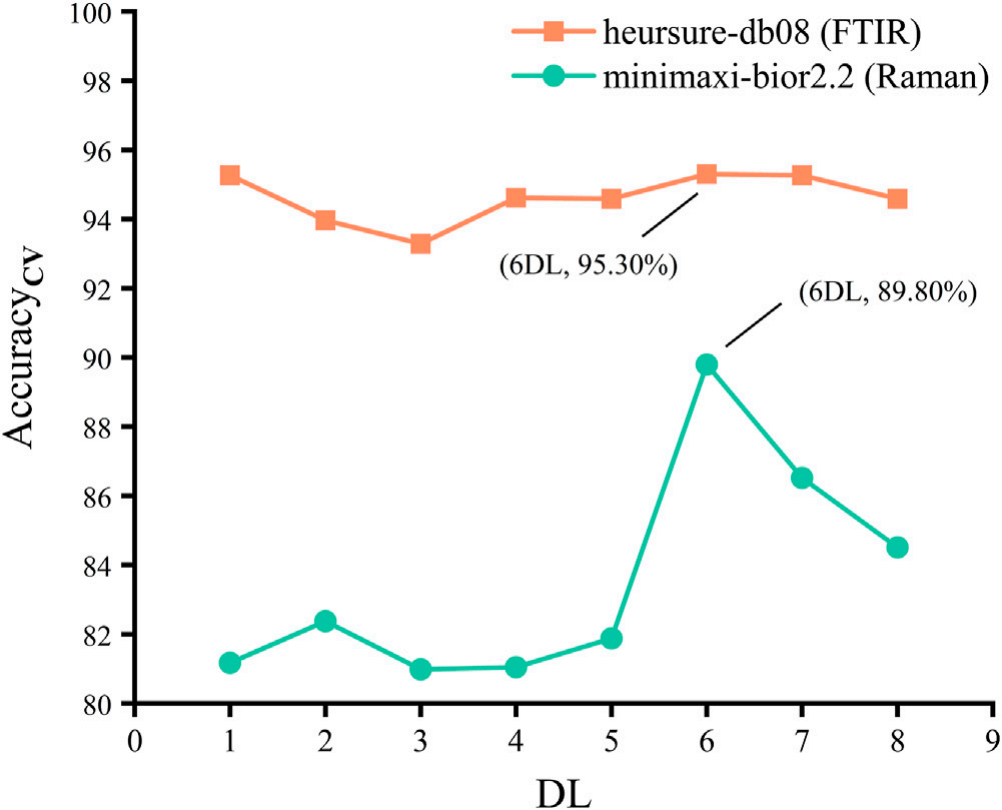
Acc_cv_ of the PLS-DA model using FTIR and Raman spectral data processed by WTD in different DLs.

Figure 3 shows the Acc_cv_ (mean value + error bar) of the PLS-DA model using Raman spectral data processed by WTD in four thresholds. It is shown that the combination of minimaxi and bior2.2 (minimaxi-bior2.2) has higher Acc_cv_ than the combination of other thresholds and wavelet functions (Figure 3D). Figure 4 shows that DL = 6 is the best DL for WTD (minimaxi-bior2.2) of Raman spectral data. Therefore, minimaxi-bior2.2 and DL = 6 were selected as the optimal wavelet parameters for WTD of Raman spectral data.

#### Comparison of Wavelet Threshold Denoising With Other Preprocessing Methods

After obtaining the optimal wavelet parameters, the spectral data processed by WTD and other preprocessing methods were analyzed with the PLS-DA model. Table 3 and Table 4 show the accuracy, specificity, and sensitivity of FTIR and Raman spectral data in the PLS-DA model, respectively. Compared with the original spectral data and the data processed by other preprocessing methods, the spectral data processed by WTD, especially the Raman spectral data, obtained a good performance in the PLS-DA model.

**TABLE 3:** Model performances of the PLS-DA model using FTIR spectral data with different preprocessing methods. (The best processing method for the PLS-DA model are presented in bold).

Method	Calibration			Prediction		
	Acc_cv_ (%)	Spe_cv_ (%)	Sen_cv_ (%)	Acc_p_ (%)	Spe_p_ (%)	Sen_p_ (%)
No	93.26	96.78	86.51	94.95	98.31	90.00
Normalization	94.62	97.80	88.34	93.94	98.31	87.50
SNV	94.65	100.00	85.56	97.98	100.00	95.00
SG	93.91	96.73	89.09	94.95	98.31	90.00
SG + Normalization	93.94	98.98	84.61	96.97	96.61	97.50
SG + Normalization + SNV	93.91	99.16	81.30	94.95	100.00	84.50
FD	92.55	98.41	79.59	92.93	100.00	82.50
FD + Normalization	87.23	94.62	73.66	89.90	96.61	80.00
FD + Normalization + SNV	87.20	95.49	72.24	91.92	96.61	85.00
SD	77.68	75.17	85.20	79.80	72.80	90.00
SD + Normalization	77.74	81.59	69.99	81.82	86.44	75.00
SD + Normalization + SNV	78.39	82.44	74.94	83.84	88.14	77.50
**WTD**	**95.30**	96.76	**92.36**	**94.95**	**94.92**	**95.00**
WTD + Normalization	93.23	98.81	82.63	94.95	100.00	87.50
WTD + Normalization + SNV	95.27	97.08	92.36	95.96	94.92	97.50

No: No preprocessing; SNV: standard normal variate; SG: Savitzky–Golay filter; FD: first derivative; SD: second derivative; WTD: wavelet threshold denoising.

The meaning of the bold values is the best processing method for PLS-DA model.

**TABLE 4:** Model performances of the PLS-DA model using Raman spectral data with different preprocessing methods. (The best processing method for the PLS-DA model are presented in bold).

Method	Calibration			Prediction		
	Acc_cv_ (%)	Spe_cv_ (%)	Sen_cv_ (%)	Acc_p_ (%)	Spe_p_ (%)	Sen_p_ (%)
No	82.38	58.65	75.09	69.70	49.02	91.67
Normalization	85.13	91.13	74.60	72.73	76.47	68.75
SNV	83.83	85.59	80.29	69.70	64.71	75.00
SG	83.02	60.99	68.46	67.68	47.06	89.58
SG + Normalization	86.43	89.39	79.76	76.77	76.47	77.08
SG + Normalization + SNV	85.78	87.47	81.91	74.75	76.47	72.92
FD	79.72	90.28	55.03	54.55	88.24	18.75
FD + Normalization	82.53	92.90	58.30	80.81	84.31	77.08
FD + Normalization + SNV	83.15	93.28	60.80	83.84	84.31	83.33
SD	74.40	68.06	88.21	65.66	58.82	72.92
SD + Normalization	81.08	91.23	58.24	71.72	76.47	66.67
SD + Normalization + SNV	80.43	90.15	58.00	70.71	70.59	70.83
**WTD**	**89.80**	90.89	**88.16**	**76.77**	**56.86**	**97.92**
WTD + Normalization	89.18	93.65	80.44	69.70	64.71	75.00
WTD + Normalization + SNV	87.14	90.51	79.57	69.70	64.71	75.00

No: No preprocessing; SNV: standard normal variate; SG: Savitzky–Golay filter; FD: first derivative; SD: second derivative; WTD: wavelet threshold denoising.

The meaning of the bold values is the best processing method for PLS-DA model.

### Data Fusion Combined with Wavelet Threshold Denoising

**FIGURE 5:**
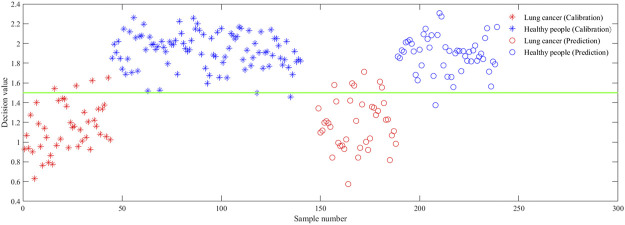
Score plot of the PLS-DA model using data fusion combined with WTD.

In order to further improve the performances of the model, data fusion was used to FTIR spectral data combined with Raman spectral data to obtain more data information. Table 5 shows the performances of the PLS-DA model using data fusion combined with different preprocessing methods. It can be seen that the data fusion has an improvement effect on each preprocessed data set. Moreover, the fusion data processed by WTD has the highest accuracy, sensitivity, and specificity in the PLS-DA model. Figure 5 shows the score plot of the PLS-DA model using data fusion combined with WTD. It can be seen that the samples from patients with lung cancer coded 1 are separated from those of healthy people coded 2 at threshold = 1.5. The PLS-DA model shows good results with 93.41% Acc_p_, 96.08% Spe_p_, and 90% Sen_p_ for the fusion data processed by WTD. The results show that FTIR spectroscopy combined with Raman spectroscopy based on data fusion and wavelet transform can effectively distinguish the serum samples of patients with lung cancer from those of healthy people.

**TABLE 5:** Performances of the PLS-DA model using data fusion combined with different preprocessing methods. (The best results for the PLS-DA model are presented in bold).

Method	Calibration			Prediction		
	Acc_cv_ (%)	Spe_cv_ (%)	Sen_cv_ (%)	Acc_p_ (%)	Spe_p_ (%)	Sen_p_ (%)
No	92.86	96.99	84.67	93.41	98.04	87.50
Normalization	93.57	98.21	83.96	91.21	94.12	87.50
SNV	94.29	95.75	86.91	91.21	94.12	87.50
SG	95.71	100.00	87.69	92.31	98.04	85.00
SG + Normalization	92.86	97.19	84.13	91.21	90.20	92.50
SG + Normalization + SNV	91.43	98.10	76.62	91.21	90.20	92.50
**WTD**	**95.00**	97.80	**90.25**	**93.41**	**96.08**	**90.00**
WTD + Normalization	92.86	94.73	87.14	81.32	80.39	82.50
WTD + Normalization + SNV	92.86	95.95	85.56	81.32	80.39	82.50

No: No preprocessing; SNV: standard normal variate; SG: Savitzky–Golay filter; WTD: wavelet threshold denoising.

The meaning of the bold values is the best processing method for PLS-DA model.

## Conclusion

Data fusion and wavelet transform were used in combination with FTIR spectroscopy and Raman spectroscopy to study the serum samples of patients with lung cancer and healthy people. The results showed that the Raman spectra of serum samples can provide more biological information than FTIR spectra of serum samples. WTD filtered the invalid information from the original spectral data, thus improving the performances of the PLS-DA model. The performance of FTIR spectral data processed by WTD in the model had higher accuracy than others. Although the addition of Raman spectral data may increase the information that is not conducive to the diagnosis of the PLS-DA model and then reduce the performance of the fusion data processed by WTD in the model, its combination with FTIR spectral data can provide better biological information. Finally, the PLS-DA model using the fusion data processed by WTD showed good results with 93.41% accuracy, 96.08% specificity, and 90% sensitivity in the prediction set, indicating that FTIR spectroscopy combined with Raman spectroscopy based on data fusion and wavelet transform could effectively distinguish the serum of patients with lung cancer from that of healthy people. In our future work, we will use richer wavelet denoising methods to improve the performance of Raman spectral data in the model, develop new methods that are more conducive to data fusion by assigning different weights to different spectral datasets, and then provide new methods for clinical screening and diagnosis of lung cancer and other diseases.

## Data Availability

The original contributions presented in the study are included in the article/Supplementary Material, further inquiries can be directed to the corresponding author.

## References

[B1] Auner G. W. Koya S. K. Huang C. Broadbent B. Trexler M. Auner Z. (2018). Applications of Raman Spectroscopy in Cancer Diagnosis. Cancer Metastasis Rev. 37 (4), 691–717. 10.1007/s10555-018-9770-9 30569241 PMC6514064

[B2] Azcarate S. M. Ríos-Reina R. Amigo J. M. Goicoechea H. C. (2021). Data Handling in Data Fusion: Methodologies and Applications. Trac Trends Anal. Chem. 143, 116355. 10.1016/j.trac.2021.116355

[B3] Bahreini M. Hosseinzadegan A. Rashidi A. Miri S. R. Mirzaei H. R. Hajian P. (2019). A Raman-Based Serum Constituents' Analysis for Gastric Cancer Diagnosis: *In Vitro* Study. Talanta 204, 826–832. 10.1016/j.talanta.2019.06.068 31357371

[B4] Baiz C. R. Błasiak B. Bredenbeck J. Cho M. Choi J.-H. Corcelli S. A. (2020). Vibrational Spectroscopic Map, Vibrational Spectroscopy, and Intermolecular Interaction. Chem. Rev. 120 (15), 7152–7218. 10.1021/acs.chemrev.9b00813 32598850 PMC7710120

[B5] Chan J. W. Taylor D. S. Zwerdling T. Lane S. M. Ihara K. Huser T. (2006). Micro-Raman Spectroscopy Detects Individual Neoplastic and normal Hematopoietic Cells. Biophysical J. 90 (2), 648–656. 10.1529/biophysj.105.066761 PMC1367069 16239327

[B6] Chavan S. S. Mahajan A. Talbar S. N. Desai S. Thakur M. D'Cruz A. (2017). Nonsubsampled Rotated Complex Wavelet Transform (NSRCxWT) for Medical Image Fusion Related to Clinical Aspects in Neurocysticercosis. Comput. Biol. Med. 81, 64–78. 10.1016/j.compbiomed.2016.12.006 28013026

[B7] Chen C. Du G. Tong D. Lv G. Lv X. Si R. (2020). Exploration Research on the Fusion of Multimodal Spectrum Technology to Improve Performance of Rapid Diagnosis Scheme for Thyroid Dysfunction. J. Biophotonics 13 (2), e201900099. 10.1002/jbio.201900099 31593625

[B8] Chen D. Shao X. Hu B. Su Q. (2004). A Background and Noise Elimination Method for Quantitative Calibration of Near Infrared Spectra. Analytica Chim. Acta 511 (1), 37–45. 10.1016/j.aca.2004.01.042

[B9] Cheng H. Xu C. Zhang D. Zhang Z. Liu J. Lv X. (2020). Multiclass Identification of Hepatitis C Based on Serum Raman Spectroscopy. Photodiagnosis Photodynamic Ther. 30, 101735. 10.1016/j.pdpdt.2020.101735 32171878

[B10] Christensen D. Rüther A. Kochan K. Pérez-Guaita D. Wood B. (2019). Whole-Organism Analysis by Vibrational Spectroscopy. Annu. Rev. Anal. Chem. 12 (1), 89–108. 10.1146/annurev-anchem-061318-115117 30978292

[B11] Comino F. Ayora-Cañada M. J. Aranda V. Díaz A. Domínguez-Vidal A. (2018). Near-infrared Spectroscopy and X-ray Fluorescence Data Fusion for Olive Leaf Analysis and Crop Nutritional Status Determination. Talanta 188, 676–684. 10.1016/j.talanta.2018.06.058 30029431

[B12] Dinç E. Yazan Z. (2018). Wavelet Transform-Based UV Spectroscopy for Pharmaceutical Analysis. Front. Chem. 6, 503. 10.3389/fchem.2018.00503 30416995 PMC6212466

[B13] Donoho D. L. Johnstone I. M. (1995). Adapting to Unknown Smoothness via Wavelet Shrinkage. J. Am. Stat. Assoc. 90 (432), 1200–1224. 10.1080/01621459.1995.10476626

[B14] Everard C. Kim M. O’Donnell C. (2016). Distinguishing Bovine Fecal Matter on Spinach Leaves Using Field Spectroscopy. Appl. Sci. 6 (9), 246. 10.3390/app6090246

[B15] Feng B. Shi H. Xu F. Hu F. He J. Yang H. (2020). FTIR-assisted MALDI-TOF MS for the Identification and Typing of Bacteria. Analytica Chim. Acta 1111, 75–82. 10.1016/j.aca.2020.03.037 32312399

[B16] Godinho M. S. Blanco M. R. Gambarra Neto F. F. Lião L. M. Sena M. M. Tauler R. (2014). Evaluation of Transformer Insulating Oil Quality Using NIR, Fluorescence, and NMR Spectroscopic Data Fusion. Talanta 129, 143–149. 10.1016/j.talanta.2014.05.021 25127577

[B17] Haware R. V. Wright P. R. Morris K. R. Hamad M. L. (2011). Data Fusion of Fourier Transform Infrared Spectra and Powder X-ray Diffraction Patterns for Pharmaceutical Mixtures. J. Pharm. Biomed. Anal. 56 (5), 944–949. 10.1016/j.jpba.2011.08.018 21873013

[B18] Jiang Y. Ma Y. (2020). Application of Hybrid Particle Swarm and Ant colony Optimization Algorithms to Obtain the Optimum Homomorphic Wavelet Image Fusion. Ann. Transl Med. 8 (22), 1482. 10.21037/atm-20-5997 33313227 PMC7729343

[B19] Lakshmi R. J. Kartha V. B. Murali Krishna C. R. Solomon J. G. Uma Devi P. Uma Devi P. (2002). Tissue Raman Spectroscopy for the Study of Radiation Damage: Brain Irradiation of Mice. Radiat. Res. 157 (2), 175–182. 10.1667/0033-7587(2002)157[0175:trsfts]2.0.co;2 11835681

[B20] Li Q. Huang Y. Zhang J. Min S. (2021). A Fast Determination of Insecticide Deltamethrin by Spectral Data Fusion of UV-Vis and NIR Based on Extreme Learning Machine. Spectrochimica Acta A: Mol. Biomol. Spectrosc. 247, 119119. 10.1016/j.saa.2020.119119 33157400

[B21] Liu L. Huan H. Li W. Mandelis A. Wang Y. Zhang L. (2021). Highly Sensitive Broadband Differential Infrared Photoacoustic Spectroscopy with Wavelet Denoising Algorithm for Trace Gas Detection. Photoacoustics 21, 100228. 10.1016/j.pacs.2020.100228 33365230 PMC7749430

[B22] Martyna A. Michalska A. Zadora G. (2015). Interpretation of FTIR Spectra of Polymers and Raman Spectra of Car Paints by Means of Likelihood Ratio Approach Supported by Wavelet Transform for Reducing Data Dimensionality. Anal. Bioanal. Chem. 407 (12), 3357–3376. 10.1007/s00216-015-8558-9 25757825

[B23] Movasaghi Z. Rehman S. Rehman I. U. (2007). Raman Spectroscopy of Biological Tissues. Appl. Spectrosc. Rev. 42 (5), 493–541. 10.1080/05704920701551530

[B24] Peng S. Chen R. Yu B. Xiang M. Lin X. Liu E. (2021). Daily Natural Gas Load Forecasting Based on the Combination of Long Short Term Memory, Local Mean Decomposition, and Wavelet Threshold Denoising Algorithm. J. Nat. Gas Sci. Eng. 95, 104175. 10.1016/j.jngse.2021.104175

[B25] Rameshnath S. Bora P. K. (2019). Perceptual Video Hashing Based on Temporal Wavelet Transform and Random Projections with Application to Indexing and Retrieval of Near-Identical Videos. Multimed Tools Appl. 78 (13), 18055–18075. 10.1007/s11042-019-7189-0

[B26] Roy I. G. (2015). On Computing First and Second Order Derivative Spectra. J. Comput. Phys. 295, 307–321. 10.1016/j.jcp.2015.04.015

[B27] Sanam T. F. Shahnaz C. (2013). Noisy Speech Enhancement Based on an Adaptive Threshold and a Modified Hard Thresholding Function in Wavelet Packet Domain. Digital Signal. Process. 23 (3), 941–951. 10.1016/j.dsp.2012.12.001

[B28] Silveira L. Jr. Sathaiah S. Zângaro R. A. Pacheco M. T. T. Chavantes M. C. Pasqualucci C. A. G. (2002). Correlation between Near-Infrared Raman Spectroscopy and the Histopathological Analysis of Atherosclerosis in Human Coronary Arteries. Lasers Surg. Med. 30 (4), 290–297. 10.1002/lsm.10053 11948599

[B29] Song D. Yu F. Chen S. Chen Y. He Q. Zhang Z. (2020). Raman Spectroscopy Combined with Multivariate Analysis to Study the Biochemical Mechanism of Lung Cancer Microwave Ablation. Biomed. Opt. Express 11 (2), 1061–1072. 10.1364/BOE.383869 32133237 PMC7041477

[B30] Stapelfeld C. Dammann C. Maser E. (2020). Sex‐specificity in Lung Cancer Risk. Int. J. Cancer 146 (9), 2376–2382. 10.1002/ijc.32716 31583690

[B31] Sudarshan V. K. Mookiah M. R. K. Acharya U. R. Chandran V. Molinari F. Fujita H. (2016). Application of Wavelet Techniques for Cancer Diagnosis Using Ultrasound Images: A Review. Comput. Biol. Med. 69, 97–111. 10.1016/j.compbiomed.2015.12.006 26761591

[B32] Sun W. Zhang X. Zhang Z. Zhu R. (2017). Data Fusion of Near-Infrared and Mid-infrared Spectra for Identification of Rhubarb. Spectrochimica Acta Part A: Mol. Biomol. Spectrosc. 171, 72–79. 10.1016/j.saa.2016.07.039 27487576

[B33] Sung H. Ferlay J. Siegel R. L. Laversanne M. Soerjomataram I. Jemal A. (2021). Global Cancer Statistics 2020: GLOBOCAN Estimates of Incidence and Mortality Worldwide for 36 Cancers in 185 Countries. CA A. Cancer J. Clin. 71, 209–249. 10.3322/caac.21660 33538338

[B34] Thakur S. K. Singh D. P. Choudhary J. (2020). Lung Cancer Identification: a Review on Detection and Classification. Cancer Metastasis Rev. 39 (3), 989–998. 10.1007/s10555-020-09901-x 32519151

[B35] Tiwari P. Viswanath S. Kurhanewicz J. Sridhar A. Madabhushi A. (2012). Multimodal Wavelet Embedding Representation for Data Combination (MaWERiC): Integrating Magnetic Resonance Imaging and Spectroscopy for Prostate Cancer Detection. NMR Biomed. 25 (4), 607–619. 10.1002/nbm.1777 21960175 PMC3298634

[B36] Wahab M. F. O'Haver T. C. (2020). Wavelet Transforms in Separation Science for Denoising and Peak Overlap Detection. J. Sep. Sci. 43 (9-10), 1998–2010. 10.1002/jssc.202000013 32108426

[B37] Xu K. Zhang C. Du T. Gabriel A. N. A. Wang X. Li X. (2021). Progress of Exosomes in the Diagnosis and Treatment of Lung Cancer. Biomed. Pharmacother. 134, 111111. 10.1016/j.biopha.2020.111111 33352449

[B38] Yan Z. Ma C. Mo J. Han W. Lv X. Chen C. (2020). Rapid Identification of Benign and Malignant Pancreatic Tumors Using Serum Raman Spectroscopy Combined with Classification Algorithms. Optik 208, 164473. 10.1016/j.ijleo.2020.164473

[B39] Yang X. Ou Q. Qian K. Yang J. Bai Z. Yang W. (2021). Diagnosis of Lung Cancer by ATR-FTIR Spectroscopy and Chemometrics. Front. Oncol. 11, 11. 10.3389/fonc.2021.753791 PMC8515056 34660320

[B40] Yang X. Ou Q. Yang W. Shi Y. Liu G. (2021). Diagnosis of Liver Cancer by FTIR Spectra of Serum. Spectrochimica Acta Part A: Mol. Biomol. Spectrosc. 263, 120181. 10.1016/j.saa.2021.120181 34311164

[B41] Zhang H. Chen C. Ma C. Chen C. Zhu Z. Yang B. (2021). Feature Fusion Combined with Raman Spectroscopy for Early Diagnosis of Cervical Cancer. IEEE Photon. J. 13 (3), 1–11. 10.1109/jphot.2021.3075958

[B42] Zhang M. Fu Z. Li G. Hou X. Lin L. (2020). Improving the Analysis Accuracy of Components in Blood by SSP-MCSD and Multi-Mode Spectral Data Fusion. Spectrochimica Acta Part A: Mol. Biomol. Spectrosc. 228, 117778. 10.1016/j.saa.2019.117778 31727519

[B43] Zhao M. Markiewicz-Keszycka M. Beattie R. J. Casado-Gavalda M. P. Cama-Moncunill X. O'Donnell C. P. (2020). Quantification of Calcium in Infant Formula Using Laser-Induced Breakdown Spectroscopy (LIBS), Fourier Transform Mid-infrared (FT-IR) and Raman Spectroscopy Combined with Chemometrics Including Data Fusion. Food Chem. 320, 126639. 10.1016/j.foodchem.2020.126639 32213423

